# Supplementary material for: Ageratina adenophora and Lantana camara in Kailash Sacred Landscape, India: Current distribution and future climatic scenarios through modeling
Source: PLoS One. 2021 May 11;16(5):e0239690. doi: 10.1371/journal.pone.0239690 (PMC8112658; doi:10.1371/journal.pone.0239690)
Supplement: S1 Appendix — (DOCX) [file pone.0239690.s001.docx]

**Appendix 1** Description of variables of three time periods used to model *Ageratina adenophora* and *Lantana camara* distribution across the KSL-India.

| Environmental variables | | | Data Source | |
| --- | --- | --- | --- | --- |
| Bioclimatic variables | | |  |  |
| Abbreviations | Description of variables | | Current | 2050 |
| BIO1 | Annual Mean Temperature | | WorldClim version 2: average monthly climate data (average for 1970-2000) | WorldClim 1.4 downscaled (CMIP5) data  (average for 2041-2060) |
| BIO2 | Mean Diurnal Range (Mean of monthly (max temp - min temp)) | |  |  |
| BIO3 | Isothermality (BIO2/BIO7) (* 100) | |  |  |
| BIO4 | Temperature Seasonality (standard deviation *100) | |  |  |
| BIO5 | Max Temperature of Warmest Month | |  |  |
| BIO6 | Min Temperature of Coldest Month | |  |  |
| BIO7 | Temperature Annual Range (BIO5-BIO6) | |  |  |
| BIO8 | Mean Temperature of Wettest Quarter | |  |  |
| BIO9 | Mean Temperature of Driest Quarter | |  |  |
| BIO10 | Mean Temperature of Warmest Quarter | |  |  |
| BIO11 | Mean Temperature of Coldest Quarter | |  |  |
| BIO12 | Annual Precipitation | |  |  |
| BIO13 | Precipitation of Wettest Month | |  |  |
| BIO14 | Precipitation of Driest Month | |  |  |
| BIO15 | Precipitation Seasonality (Coefficient of Variation) | |  |  |
| BIO16 | Precipitation of Wettest Quarter | |  |  |
| BIO17 | Precipitation of Driest Quarter | |  |  |
| BIO18 | Precipitation of Warmest Quarter | |  |  |
| BIO19 | Precipitation of Coldest Quarter | |  |  |
| Topographic variables | | | | |
| DEM | digital elevation model | Global Digital Elevation Model Version 2 (GDEM V2) | | |
| EUCL_Water | euclidean distance from primary and secondary water bodies | OpenStreetMapData (http://openstreetmapdata.com) | | |
| EUCL_Road | euclidean distance from road | OpenStreetMapData (http://openstreetmapdata.com) | | |
| Slope | Angle of slope in degree | Derivative of digital elevation model | | |
| Aspect | Slope faces in 3-dimensional space | Derivative of digital elevation model | | |


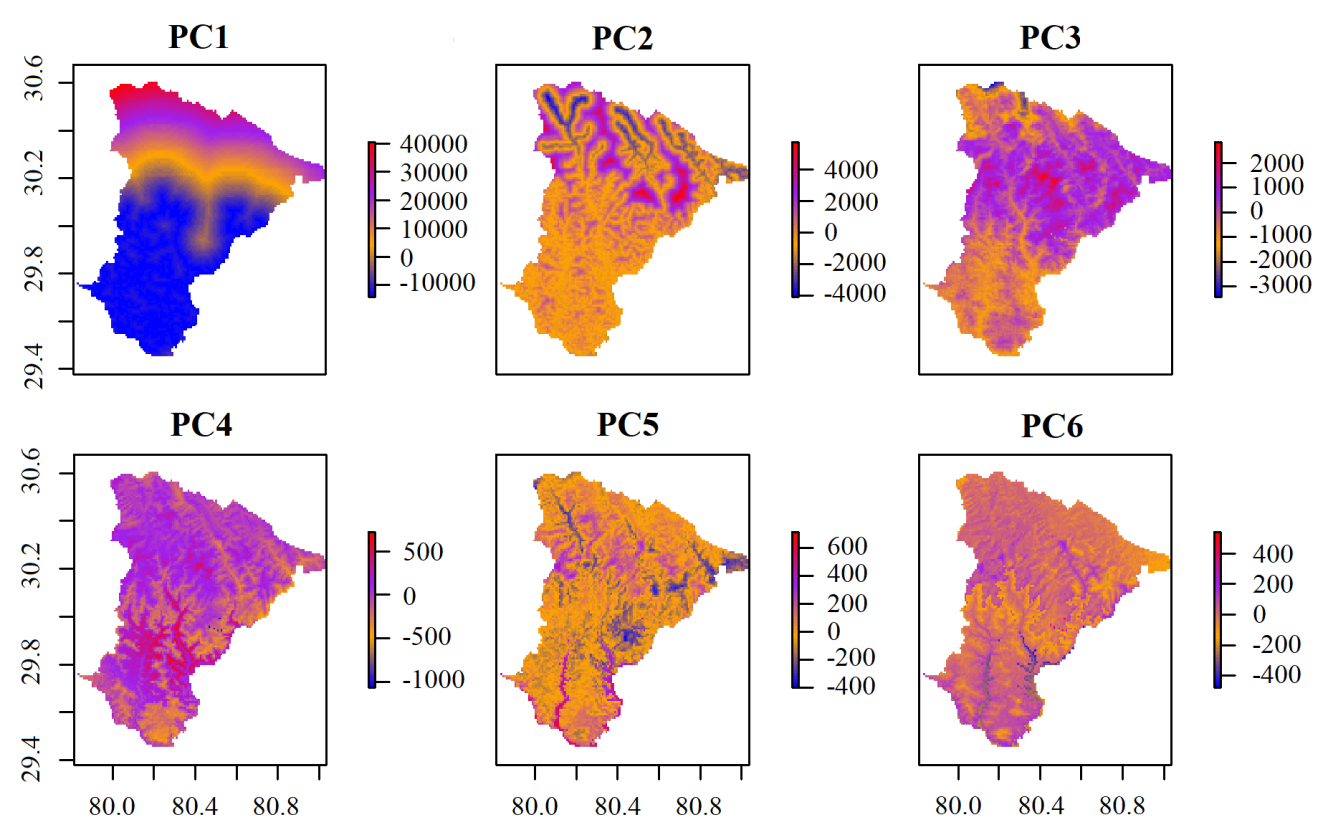


**Appendix 2** First six principal components as raster layers generated from the 24 environmental layers of current years.


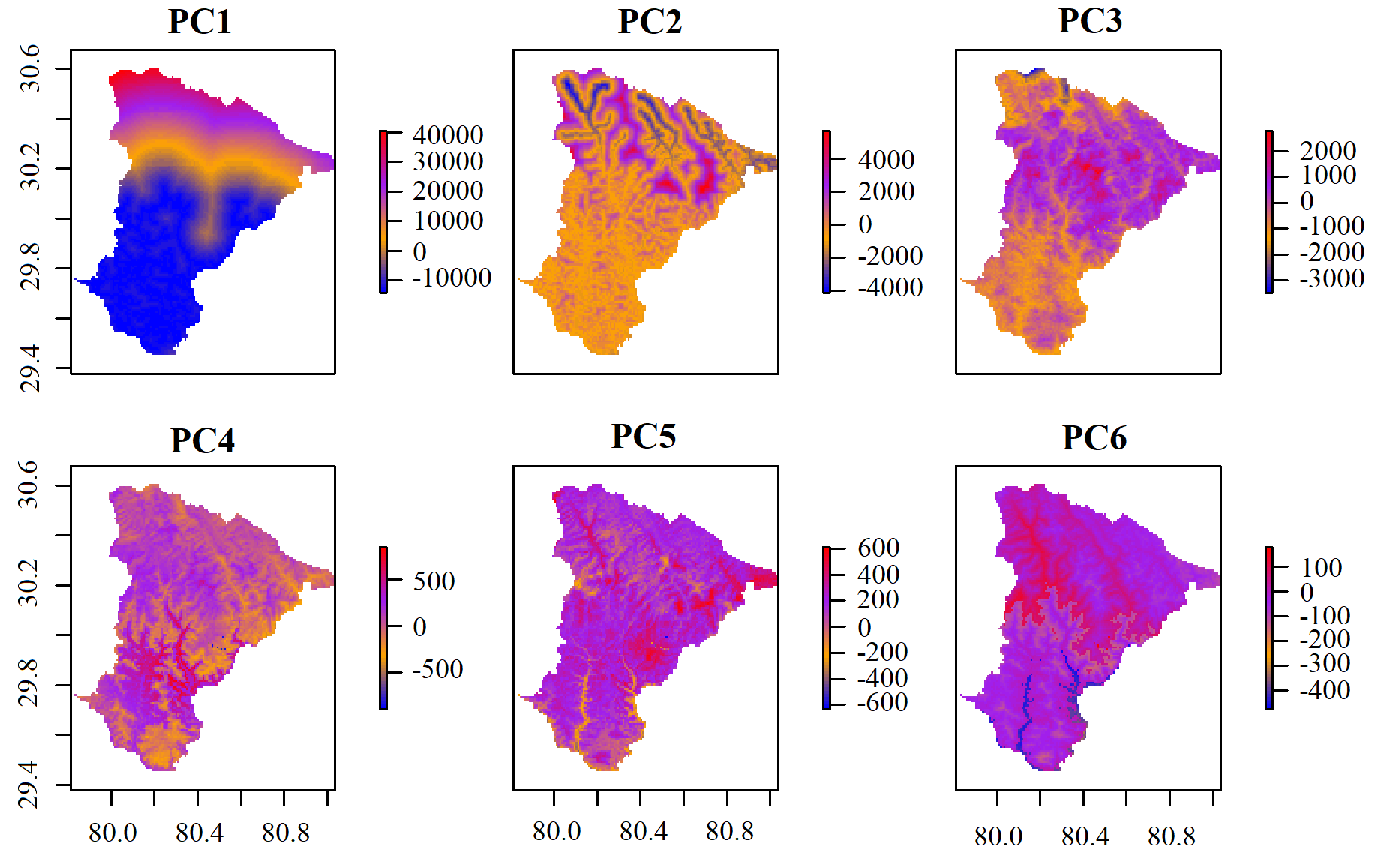


**Appendix 3** First six principal components as raster layers generated from the 24 environmental layers of 2050 years.

**Appendix 4** Results of model evaluation other than Test AUC and Training AUC.

| **Species** | **The current year** | | | | | | **Year 2050** | | | | | |
| --- | --- | --- | --- | --- | --- | --- | --- | --- | --- | --- | --- | --- |
|  | Test AUC | Training AUC | Kappa max | True Positive Rate | True Negative Rate | True Skill Statistics | Test AUC | Training AUC | Kappa max | True Positive Rate | True Negative Rate | True Skill Statistics |
| *Ageratina Adenophora* | 0.81 | 0.82 | 0.6 | 0.62 | 0.92 | 0.54 | 0.81 | 0.82 | 0.61 | 0.63 | 0.93 | 0.56 |
| *Lantana Camara* | 0.93 | 0.93 | 0.67 | 0.72 | 0.94 | 0.66 | 0.92 | 0.93 | 0.66 | 0.74 | 0.95 | 0.69 |


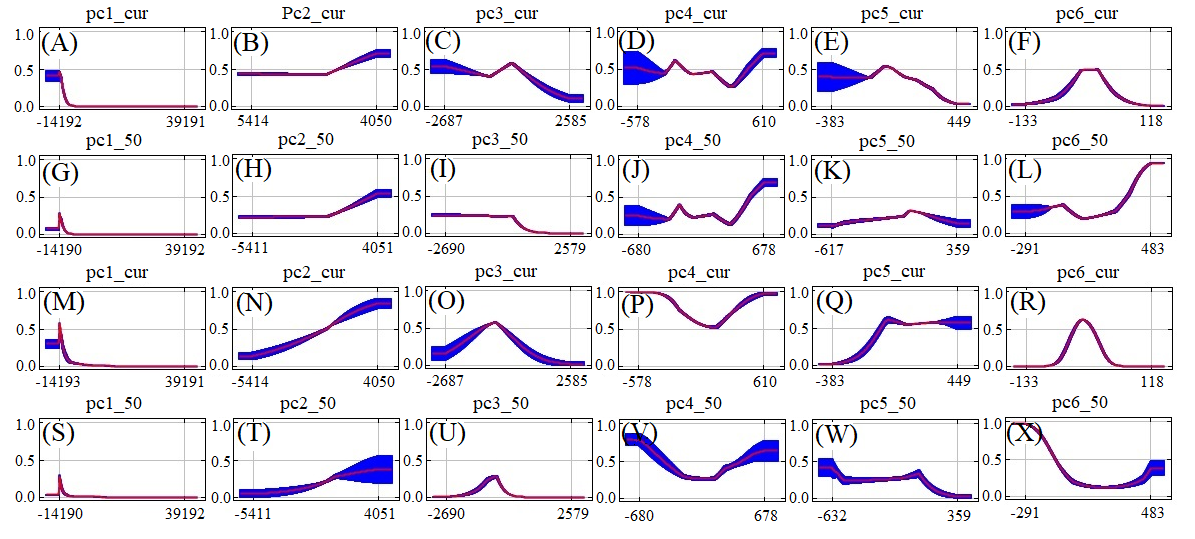


**Appendix 5** These curves show how each PCs affects the MaxEnt prediction. The curves show how the predicted probability of presence changes as each PC is varied, keeping all other environmental variables at their average sample value. The curves show the mean response of the 10 replicates of each *MaxEnt* runs. (A-F): Response curves of *Ageratina adenophora* for current year, (G-L): Response curves of *Ageratina adenophora* for the year 2050, (M-R): Response curves of *Lantana camara* for current year, (S-X) Response curves of *Lantana camara* for the year. 205
